# Neutralizing Antibody Response Characteristics in Elderly Patients with SARS-CoV-2 Infection and Their Association with Clinical Phenotypes

**DOI:** 10.3390/vaccines13111107

**Published:** 2025-10-29

**Authors:** Yunhui Li, Li Wang, Jiayue Ma, Wenqi Geng, Yajie Wang

**Affiliations:** 1 National Key Laboratory of Intelligent Tracking and Forecasting for Infectious Diseases, Beijing Key Laboratory of Viral Infectious Diseases, Department of Clinical Laboratory, Beijing Ditan Hospital, Capital Medical University, Beijing 100015, China; liyunh04@163.com (Y.L.); 18242058623@126.com (L.W.); 17716585476@163.com (J.M.); 2 Department of Clinical Laboratory, Peking University Ditan Teaching Hospital, Beijing 100015, China; 19832321808@163.com

**Keywords:** SARS-CoV-2, neutralizing antibody, the elderly

## Abstract

**Background/Objectives**: Although SARS-CoV-2 infection often follows a self-limiting course, its public health impact remains persistent. Older adults exhibit unique susceptibility to infection due to immunosenescence. Therefore, in order to offer recommendations for improving management options for older persons, this study intends to examine the immunological properties of NAb in the elderly population. **Methods**: Elderly patients aged 60 years and older infected during the prevalence of BF.7 and EG.5 variants were enrolled. The patterns of NAb responses in infected patients under both natural and vaccine-induced immunity were explored using bead-based proteomics techniques. The associations between NAb and IgG antibody levels, clinical characteristics, and traditional inflammatory indicators were evaluated using systematic analysis. Based on NAb levels, SARS-CoV-2 strains were immunologically classified. **Results**: There was a positive correlation between the severity of the disease and the strength of the NAb response. Because of more extensive immune activation, severe instances in elderly patients showed higher levels of NAb responses. When compared to the uninfected group, people who had received two doses of vaccination exhibited greater NAb levels. Additionally, there was a link between NAb and IgG levels, but as the virus evolved, this correlation progressively diminished. Three serotypes of SARS-CoV-2 were identified based on NAb response characteristics: pre-Omicron, Omicron, and XBB serotypes. **Conclusions**: The results show the features of NAb responses in older patients, which could help with the creation of future vaccines and public health initiatives.

## 1. Introduction

Since its emergence in December 2019, Severe Acute Respiratory Syndrome Coronavirus 2 (SARS-CoV-2) has led to a global pandemic. As of 1 July 2025, more than 770 million confirmed cases and over 7 million deaths have been reported worldwide (https://covid19.who.int/; accessed on 11 October 2020).

Prospective cohort research found that non-hospitalized symptomatic patients often report less daily activities because of prolonged respiratory and systemic symptoms, despite the fact that the majority of illnesses present with asymptomatic or moderate self-limiting courses [1]. A significant socioeconomic burden is also associated with this, which includes indirect production losses, healthcare utilization, and missed work. These results demonstrate the ongoing effects of SARS-CoV-2 infection on public health.

Viral evolution and host immune responses interact dynamically to drive the continuous spread of SARS-CoV-2. According to available data, the receptor-binding domain (RBD) of the viral spike (S) protein confers a transmission advantage through two key mechanisms [2]: first, by improving its binding affinity to the host angiotensin-converting enzyme 2 (ACE2), which increases infectivity; and second, by promoting immune evasion, which weakens immunity gained from prior infection or immunization by decreasing the effectiveness of neutralizing antibodies (NAbs). NAbs, as key effector molecules produced by B cells in response to viral infection or vaccination, play a crucial role in humoral immunity [3]. Most NAbs targeting SARS-CoV-2 are directed against the S protein and its functional domains, exerting antiviral activity through diverse molecular mechanisms.

The elderly exhibit heightened susceptibility to infections due to immunosenescence [4], manifested as ineffective T cell priming, loss of naive T cell diversity, decreased antibody maturation, and/or impaired memory. According to epidemiological data, people aged 65 years and older had a markedly higher chance of developing serious illness, contracting a secondary infection, and dying after contracting SARS-CoV-2 [5]. Older people frequently show blunted humoral immune responses, such as lower antibody titers, reduced antibody affinity maturation, and attenuated T-cell activity [6,7], as well as shorter-lived immunity (e.g., a higher probability of NAb levels declining below the seropositivity threshold within six months after the second vaccine dose) [8], even though vaccination partially mitigates these immune deficiencies. As a result, this group is still susceptible to breakthrough infections and worsening clinical consequences [9].

Unfortunately, the majority of recent studies on SARS-CoV-2 infection in older persons have concentrated on clinical phenotypic characterization, while the relationship between dynamic NAb responses and clinical outcomes has not been sufficiently investigated. We previously developed a fluorescence-encoded magnetic bead-based high-throughput broad neutralizing-antibody (bNAb) test [10]. This platform has good repeatability, enables simultaneous detection of NAbs against various SARS-CoV-2 variants, and accurately assesses NAb-mediated immune protection [11]. In this study, we enrolled patients aged 60 years and older with primary SARS-CoV-2 infection to systematically analyze the relationships between NAb levels and viral variant characteristics, immunization history, and clinical classifications. We aimed to elucidate the biological features of NAb responses in the elderly and evaluate their prognostic significance, thereby providing a scientific foundation for optimizing stratified management, including early intervention in high-risk groups and tailored vaccination strategies in this vulnerable population.

## 2. Materials and Methods

### 2.1. Samples and Patients

Patients were selected from our previously published cohort [11]. Specifically, we included patients aged 60 years and older from the BF.7 wave (December 2022–March 2023, *n* = 65) and the EG.5 wave (July–September 2023, *n* = 57), resulting in a total of 122 patients for this analysis. Accordingly, the study comprised two distinct groups: the BF.7 infection group and the EG.5 infection group. Most vaccine recipients had received inactivated virus vaccines (CoronaVac or BBIBP-CorV), which are widely used in China. Disease severity was classified based on China National Health Commission Guidelines for Diagnosis and Treatment of SARS-CoV-2 infection (Trial Version 10).

(1) Mild: Characterized primarily by upper respiratory tract symptoms such as dry throat, sore throat, cough, fever, etc.;
(2) Moderate: (i) Defined by persistent high fever (>3 days) and/or respiratory symptoms including cough and shortness of breath, with respiratory rate (RR) < 30 breaths/min and oxygen saturation > 93% at rest. (ii) Radiological imaging reveals characteristic presentations of COVID-19 pneumonia infection.
(3) Severe: An adult meeting any of the following criteria (not explained by other causes): (i) Shortness of breath with RR ≥ 30 breaths/min; (ii) Oxygen saturation ≤ 93% at rest; (iii) Arterial partial pressure of oxygen (PaO2)/fraction of inspired oxygen (FiO2) ≤ 300 mmHg (1 mmHg = 0.133 kPa); (iv) Progressive clinical worsening with radiographic evidence of >50% lung lesion progression within 24–48 h.
(4) Critical: Meeting any of the following: (i) Respiratory failure requiring mechanical ventilation; (ii) Shock; (iii) Multiple organ dysfunction requiring ICU monitoring and treatment.

All the infection samples were confirmed via polymerase chain reaction (PCR) or antigen testing.

This study was reviewed and approved by the Institutional Review Board of the Ethics Committee of Ditan Hospital (No. DTEC-KY2022–052-02 and No. DTEC-KY2021–010-01). All procedures performed in this study were in accordance with the ethical standards of the Declaration of Helsinki.

### 2.2. Detection of NAbs in Experimental Samples

The method for NAb detection has been previously described in detail [11]. Briefly, we applied a high-throughput bNAb assay based on fluorescence-encoded magnetic beads (Shenzhen Wellgrow Technology Co., Ltd., Beijing, China) and a flow cytometer (Shenzhen Wellgrow Technology Co., Ltd., Beijing, China) to systematically evaluate the characteristics of specific immune responses to various SARS-CoV-2 variants following natural infection and vaccination. These variants included six pre-Omicron variants (D614G, Alpha, Beta, Gamma, Delta, Kappa) and twelve Omicron sublineages (BA.1, BA.2, BA.2.75, BA.3, BA.4, BA.5, BF.7, BQ.1.1, XBB, XBB.1.5, XBB.1.9.1, and EG.5.1).

Trimerized Spike protein (Sino Biological and ACROBiosystems, Beijing, China) from six pre-Omicron variants and twelve Omicron variants were coupled to fluorescence-encoded magnetic beads. Serum samples were mixed and incubated with the conjugated magnetic beads, allowing NAbs to bind to the RBD of the S protein, thereby blocking its interaction with biotinylated ACE2 receptors (Sino Biological, Beijing, China). Subsequently, streptavidin–phycoerythrin (SA-PE) (Thermo Fisher Scientific, Waltham, MA, USA) was used as a fluorescent probe for labeling. The median fluorescence intensity (MFI) was measured via flow cytometry to quantitatively assess the neutralization capacity of NAbs based on their inhibition of S–ACE2 binding.

To quantify the analytical results, neutralization rate calculation formula was established:

Neutralization (%) = [1 − (XS/X0)] × 100%

X0 represents the MFI of the blank well, which reflects the fluorescence signal resulting from S protein–ACE2 binding in the absence of NAbs.

XS represents the MFI of the sample well, representing the fluorescence signal after the inhibition of S protein–ACE2 binding by NAbs.

The neutralization (%) is positively correlated with the level of NAbs. When the NAb level in the sample is high, the inhibition of S-ACE2 binding is stronger, resulting in a lower MFI value and a higher neutralization (%). Conversely, when the NAb level is low, the inhibition of S-ACE2 binding is weaker, resulting in a higher MFI value and a lower neutralization (%). Thus, this analytical method enables precise quantification of NAb efficacy, providing reliable technical support for experimental research and clinical applications.

### 2.3. Statistical Analysis

The raw neutralization rates for each variant were first normalized to a 0–1 range using min-max normalization. Outliers with absolute Z-scores greater than 3 were then excluded. The normalized neutralization rates were subsequently used for all further analyses [11]. To thoroughly characterize NAb responses in elderly individuals infected with SARS-CoV-2 across various viral variants, we employed Z-score transformation to standardize the experimental data. Subsequently, a multi-variant NAb response profile was constructed. Cluster analysis was performed to visualize variant-specific response patterns through a heatmap.

All statistical analyses were performed using R 4.3.3 software. The normality of continuous variables was assessed with the Shapiro–Wilk test. Non-normally distributed data were summarized as median and interquartile range (IQR). A small number of missing values were imputed using median substitution. The comparisons between two independent groups for continuous variables were performed using the Wilcoxon rank-sum test, while the Pearson’s chi-squared test was used for categorical variables. For comparisons of continuous variables across more than two groups, the Kruskal–Wallis test was applied.

Correlations between neutralizing antibodies and laboratory parameters were visualized using the chordDiagram function from the circlize package. Cluster analysis is carried out automatically by the pheatmap() function. The association between NAbs and IgG antibodies was evaluated using Spearman’s rank correlation. A *p*-value less than 0.05 was considered statistically significant.

## 3. Results

### 3.1. Demographic and Clinical Data of the Enrolled Patients

This study included 122 patients over the age of 60 with primary SARS-CoV-2 infection. Participants were separated into two groups based on the infection waves caused by different variants: the BF.7 infection (*n* = 65) and the EG.5 infection (*n* = 57). Patients infected with BF.7 were enrolled in December 2022–March 2023, during which BF.7 was the predominant circulating variant in the region [12]. Those in the EG.5 infection group were admitted between July and September 2023, when EG.5 and its sublineages were the dominant local strains [13].

Patients were stratified according to pre-infection vaccination status into the following subgroups: unvaccinated (0), one dose (1), two doses (2), three doses (3), four doses (4), and “unknown” group owing to unreliable recall or missing records. The duration of infection was defined as the period from the symptom onset to the sample collection. The demographic and clinical characteristics of the infected individuals are detailed in Table 1. Specifically, the BF.7 infection group comprised 43 males (66.2%) and 22 females (33.8%), with a median age of 70.0 years (IQR: 65.0–74.0) and a median infection duration of 15 days (IQR: 13–20). The EG.5 infection group included 26 males (45.6%) and 31 females (54.4%), with a median age of 72.0 years (IQR: 68.0–80.0) and a median infection duration of 15 days (IQR: 7–18).

### 3.2. Characteristics of NAb Response in Elderly Infected Patients

To investigate the association between NAb responses and disease severity among elderly individuals diagnosed with SARS-CoV-2 infection, as well as to assess its clinical predictive value, this study categorized 65 patients infected with the BF.7 variant into the non-severe group (comprising mild and moderate cases, *n* = 34) and the severe group (including severe and critical cases, *n* = 31). Disease severity classification was evaluated according to China National Health Commission Guidelines for Diagnosis and Treatment of SARS-CoV-2 infection (Trial Version 10). Similarly, 57 patients infected with the EG.5 variant were divided into the non-severe group (mild and moderate cases, *n* = 46) and the severe group (severe and critical cases, *n* = 11). The potential of NAb responses in predicting disease progression was systematically evaluated.

**Figure 1:**
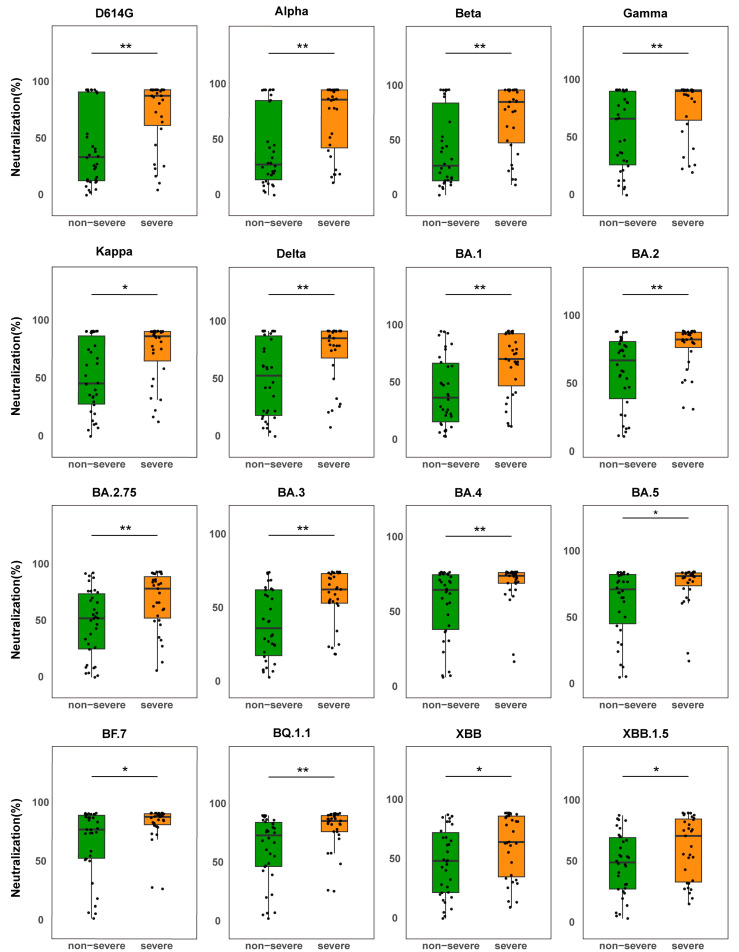
Comparison of NAb levels between severe and non-severe BF.7-infected patients. Box plots show NAb levels against the SARS-CoV-2 variant S protein in non-severe patients (green, *n* = 34) and severe patients (orange, *n* = 31). The boxes represent the median (center line) and interquartile range (25–75%). * *p* < 0.05, ** *p* < 0.01.

According to statistical analysis, the severe group of the BF.7 infection cohort had considerably greater NAb levels against important variants than the non-severe group (*p* < 0.05) (Figure 1), demonstrating both broad-spectrum variant coverage and strong NAb responses. These findings imply that severe infection in older people is linked to a more acute NAb response, which may be caused by stronger immunological activation.

Interestingly, the median NAb levels in the severe group were considerably higher than those in the non-severe group (*p* < 0.05) even though they were lower against XBB and XBB.1.5 variations than against the ancestral strain (e.g., D614G). This suggests that individuals with severe disease still have a greater total NAb response, even when the cross-neutralizing potential against variations may be partially compromised due to antigenic shift. The small sample size in the severe group (*n* = 11) may have contributed to the lack of a statistically significant difference in NAb levels between groups in the EG.5-infected cohort (*p* > 0.05) (Figure S1). Subgroup studies revealed that, in contrast to the non-severe group, the severe group had greater median NAb levels against a number of variations, including BA.5, BF.7, BQ.1.1, XBB, XBB.1.5, XBB.1.9.1, and EG.5.1.

All things considered, these results indicate that the correlation between disease severity and NAb response strength in the elderly may be variant-dependent. In this study, a strong correlation was observed in BF.7-infected patients, whereas this association was less pronounced in the EG.5-infected cohort.

### 3.3. Impact of Vaccination on NAb Responses in Elderly Infected Patients

**Figure 2:**
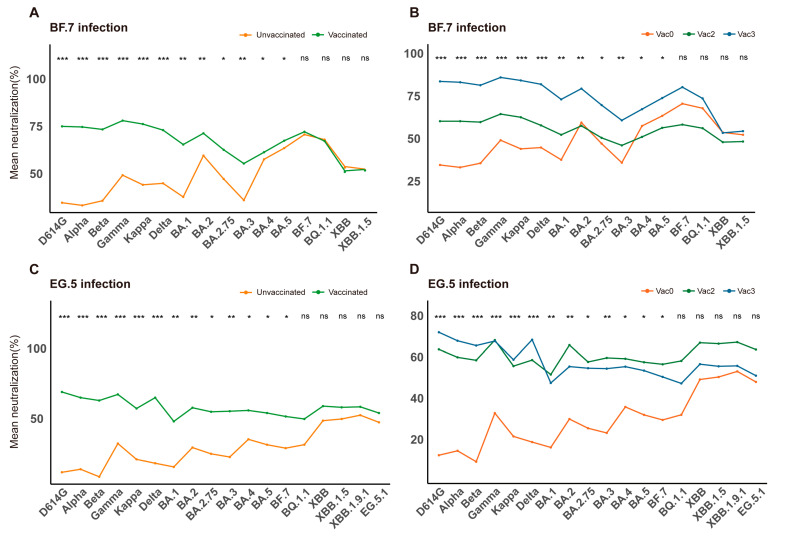
Impact of vaccination on NAb response. NAb responses were analyzed according to vaccination status. (**A**) Comparison of NAb levels between BF.7 breakthrough infection (unvaccinated, red) and natural infection (vaccinated, green). (**B**) Comparison of NAb levels among elderly individuals infected with the BF.7 variant after different vaccination regimens. (**C**) Comparison of NAb levels between EG.5 breakthrough infection (vaccinated, red) and natural infection (unvaccinated, green). (**D**) Comparison of NAb levels among elderly individuals infected with the EG.5 variant after different vaccination regimens. Vac0: Unvaccinated group (red); Vac2: Two-dose vaccination group (green); Vac3: Three-dose vaccination group (blue). * *p* < 0.05, ** *p* < 0.01, *** *p* < 0.001; ns: not significant.

Based on vaccination history, patients were categorized into unvaccinated, one-dose, two-dose, and three-dose groups. When compared to the unvaccinated group, vaccination dramatically raised NAb levels in the BF.7-infected cohort (Figure 2A). The three-dose group showed a stronger NAb response than the two-dose group, although the difference did not reach statistical significance (Figure 2B), possibly due to heterogeneity in immune responses among the elderly. In the EG.5-infected cohort, the dose-dependent effect (three doses vs. two doses) was not significant (Figure 2D), while the median NAb levels in the vaccinated groups were considerably greater than those in the unvaccinated group (Figure 2C). However, these findings demonstrated that vaccination produces measurable immunogenicity and can elicit strong NAb responses in this demographic.

Interestingly, there was no statistically significant difference in NAb levels between the groups that received the vaccination and those that did not (*p* > 0.05) against XBB and its sublineages, such as XBB.1.5, XBB.1.9.1, and EG.5.1. This suggests that previous COVID-19 vaccination was ineffective at eliciting protective immunity against the XBB variant series. The idea that the XBB variants show notable antigenic diversity from earlier strains is supported by this serological observation [14].

To assess the association between vaccination and disease severity, we analyzed all patients with a documented vaccination history from the BF.7 and EG.5 infection cohorts. Participants were classified into severe (*n* = 31) and non-severe (*n* = 60) groups based on clinical severity. The comparison of characteristics between severe and non-severe groups is detailed in Supplementary Table S1. Differences in age, sex, vaccination status, and comorbidities between the groups were evaluated. A comorbidity was defined as the presence of at least one of the following: diabetes, hypertension, coronary heart disease, or malignancy. Univariate analysis identified older age, male sex, and comorbidities as risk factors for severe disease. In a multivariate logistic regression model adjusted for age, sex, comorbidities, and disease duration, vaccination showed a trend toward a 19.4% reduction in the risk of severe disease (OR = 0.806). However, this association was not statistically significant (95% CI: 0.300–2.175, *p* > 0.05). One plausible explanation is that a moderate protective effect may exist but was not detectable due to the limited sample size, which likely reduced the statistical power to distinguish this effect from chance.

### 3.4. Correlation Analysis Between NAb and Laboratory Indicators

To further enhance clinical management strategies for elderly patients, this study investigated the association between NAb levels, inflammatory cytokines, and clinical manifestations.

**Figure 3:**
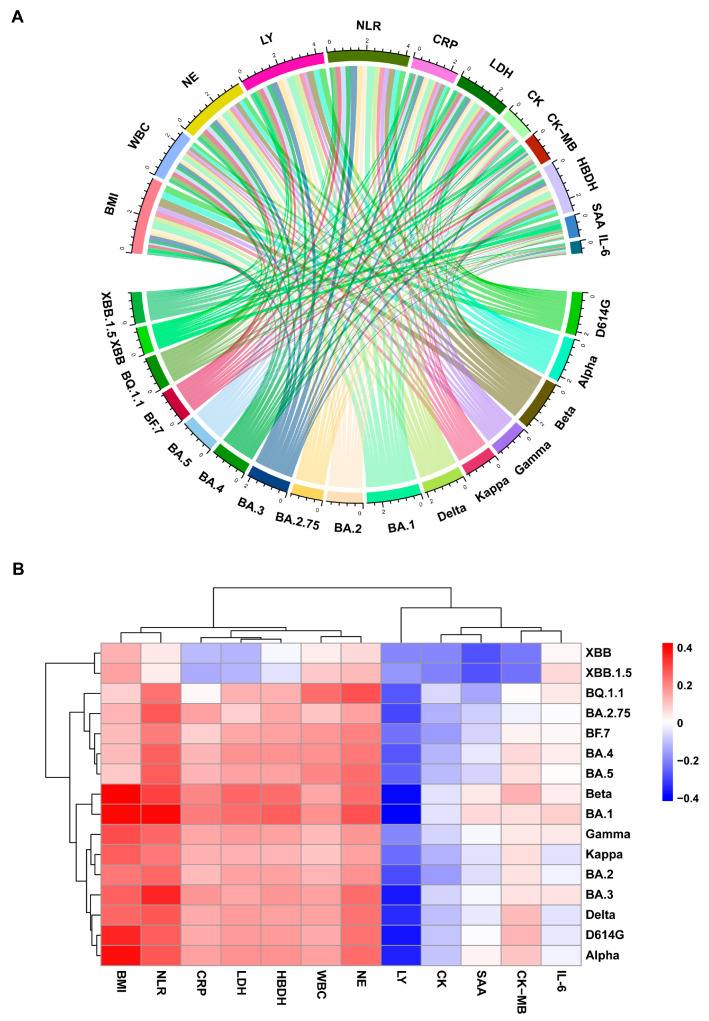
Correlation analysis between NAbs and laboratory parameters in the BF.7 infection cohort. (**A**) Chord diagram showing correlations between variant-specific NAbs and lab parameters. The top and bottom arcs represent laboratory parameters and variant-specific NAb responses, respectively. (**B**) Heatmap of Spearman’s correlation coefficients between NAbs against different variants and laboratory parameters. Blue denotes negative correlations; red indicates positive correlations.

The findings indicated significant associations between NAb levels and various indicators, including body mass index (BMI), white blood cell count (WBC), neutrophil count (NE), lymphocyte count (LY), neutrophil-to-lymphocyte ratio (NLR), C-reactive protein (CRP), lactate dehydrogenase (LDH), and hydroxybutyrate dehydrogenase (HBDH) (Figure 3A). Specifically, NAb levels exhibited positive correlations with BMI, WBC, NE, NLR, CRP, LDH, and HBDH, while a weak negative correlation was observed with LY (Figure 3B). Although these associations did not reach statistical significance, the overall trends suggest a potential state of “inflammatory activation–lymphocyte exhaustion” in elderly individuals [15].

Additionally, this study has established a positive correlation between NAb and BMI, indicating heightened humoral immune responses in obese elderly patients due to chronic inflammatory resulting in elevated NAb levels. WBC, NE, and NLR are conventional markers of the inflammatory response, with elevated levels indicating an innate immune response dominated by neutrophils. Meanwhile, CRP, LDH, and HBD reflect the degree of systemic inflammation, tissue damage, and cellular metabolic status. The observed positive correlation trends between NAb and these indicators suggest that NAb levels may fluctuate in conjunction with the systemic inflammatory condition, potentially associated with B-cell hyperactivation during the post-viral “cytokine storm”.

### 3.5. Correlation Analysis Between NAb and IgG Antibody Responses

**Figure 4:**
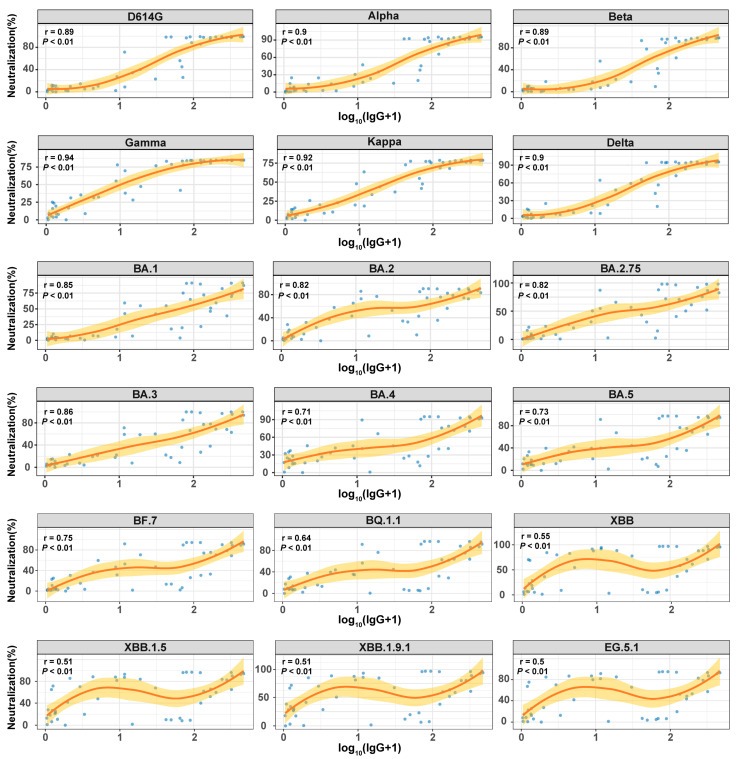
Correlation between IgG and NAb in the EG.5 infection cohort. Spearman correlation analysis was used to evaluate the relationship between IgG antibodies (log_10_(SNR+1) transformed values, x-axis) and NAbs (neutralization rate %, y-axis). The scatter plot displays serum samples (blue dots), with the red curve representing the non-linear association trend.

Considering the impact of SARS-CoV-2 variant evolution on humoral immune responses, we analyzed the relationship between IgG antibody levels and NAb levels. Patients from the EG.5 infection cohort were analyzed for serum IgG levels targeting the ancestral strain using a commercial chemiluminescence immunoassay kit. Functional NAbs against S proteins of various variants were assessed through fluorescence-encoded magnetic bead-based proteomic technology. A systematic correlation analysis between IgG and NAb levels revealed a significant positive relationship (Spearman r = 0.50–0.94, *p* < 0.01; Figure 4), suggesting a synergistic effect between IgG’s antigen-binding capacity and NAb’s functional neutralization within the humoral immune response. Subsequent sub-analysis, stratified by viral sublineage, indicated variability in the strength of the NAb–IgG correlation based on antigenic evolution.

For previous variants (such as D614G, Alpha, Beta, Gamma, Kappa, and Delta), NAb levels displayed a robust correlation with IgG levels (Spearman r = 0.89–0.94, *p* < 0.01). This strong association can be attributed to the structural conservation of the S protein, especially within the RBD, across these earlier strains. The epitopes recognized by IgG assays designed against the original strain remained largely conserved among these variants. Consequently, serum IgG antibodies maintained their capacity to efficiently bind viral antigens, leading to elevated IgG levels and frequently neutralized key epitopes crucial for viral entry, resulting in increased NAb levels. This dual action facilitated a high level of agreement between these two immune parameters.

In contrast, for later emerging strains like XBB, XBB.1.5, XBB.1.9.1, and EG.5.1, the correlation between NAb and IgG was less robust (Spearman r = 0.50–0.55, *p* < 0.01). This discrepancy could be attributed to significant antigenic drift, characterized by numerous mutations in the RBD that have accumulated in Omicron subvariants [16,17]. These mutations induce changes in the spatial conformation of critical antigenic epitopes. Consequently, assays detecting IgG based on the original strain may still demonstrate partial affinity to viral antigens, potentially yielding elevated IgG levels, while the actual neutralizing capacity is substantially diminished. As a result, the correlation between IgG and NAb levels weakens. A notable portion of these IgG antibodies may lack neutralizing capabilities or have reduced potency, leading to a noticeable disconnection between quantifiable IgG levels and effective neutralization.

### 3.6. Cluster Analysis Based on NAb Response Profiles

**Figure 5:**
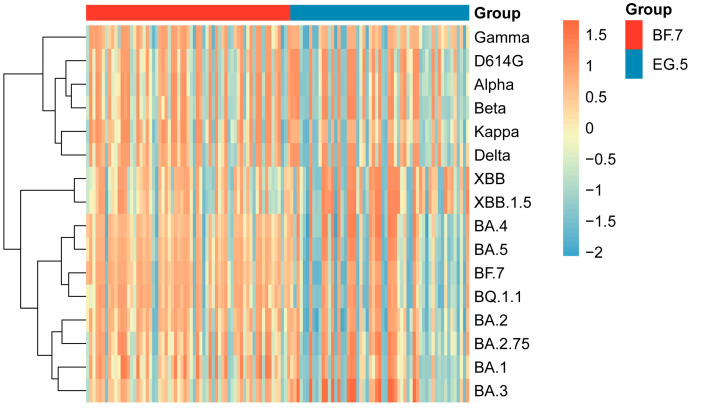
Clustering analysis of SARS-CoV-2 based on NAb responses. Clustering heatmap of NAb responses in the infection cohort. Based on Z-score normalized NAb data, the heatmap visually represents the intensity of NAb responses against different SARS-CoV-2 variants in the infected population through clustering analysis.

Next, by employing NAb data from all cohorts, NAb response profile was constructed to visualize variant-specific response patterns (Figure 5). Cluster analysis of NAb levels indicated that pre-Omicron variants (Gamma, D614G, Alpha, Beta, Kappa, and Delta) clustered together due to their highly similar NAb response profiles. In contrast, Omicron variants (BA.1, BA.2, BA.2.75, BA.3, BA.4, BA.5, BF.7, BQ.1.1, XBB, and XBB.1.5) were distinctly separated from pre-Omicron strains, forming a separate cluster. Further examination of Omicron sublineages revealed that XBB strains (including XBB and XBB.1.5) displayed sub-clustering within the Omicron cluster, indicating antigenic divergence from other Omicron variants and suggesting the emergence of a distinct serological phenotype.

BF.7 and BQ.1.1 were grouped in a common sub-cluster, while BA.4 and BA.5 formed a distinct cluster, consistent with known evolutionary relationships of SARS-CoV-2. These results offer serological evidence supporting the proposed evolutionary path of SARS-CoV-2 variants and highlight the intricate relationship between NAb responses and viral antigenic changes.

## 4. Discussion

As emerging SARS-CoV-2 variants continue to pose challenges to global public health, understanding the humoral immune response in vulnerable elderly populations becomes critical. This study aimed to systematically evaluate the NAb responses in elderly patients infected with predominant variants, with a focus on their association with clinical phenotypes.

First, our data confirm that advanced age is a risk factor for greater disease severity. In the BF.7 infection cohort, patients with severe COVID-19 exhibited significantly higher neutralizing antibody (NAb) levels against predominant variants compared to non-severe cases (Figure 1). This positive association between disease severity and enhanced neutralizing activity suggests that a robust antibody response may reflect pathological immune activation rather than confer protection, particularly in vulnerable populations [11,18].

The WHO recommends evaluating the immunogenicity and efficacy of vaccines in the elderly population. An ideal vaccine should elicit a protective immune response after one or two doses, even in vulnerable groups such as the elderly or immunocompromised individuals. This is particularly critical for the primary vaccination of these populations, as they face a higher risk of severe outcomes following infection [19]. Vaccination can significantly reduce symptomatic COVID-19 in older adults and further prevent the occurrence of severe disease [20]. A prospective study indicated that vaccination provides protection for the elderly and reduces SARS-CoV-2 transmission [21]. Within the specific context of immunosenescence and the high prevalence of comorbidities in the elderly, our study suggests that vaccination may play a beneficial role in mitigating the risk of severe disease; however, limitations such as the small sample size precluded statistically robust evidence. Future research should aim to validate these findings in the local population through larger sample sizes and prospective designs.

Compared to the unvaccinated group, the elderly who received two-dose vaccine exhibited significantly higher NAb levels. Thus, preventive measures in this population should emphasize infection prevention through the completion of primary immunization. Promoting vaccination to establish an immune protection remains essential. Nonetheless, NAbs induced by vaccines based on the ancestral strain exhibit a significantly reduced cross-neutralizing capacity against later Omicron sublineages such as BF.7 and EG.5. In this study, there was no statistical differences in the levels of NAb between vaccinated and unvaccinated patients when tested against their own infecting variants. The observed phenomenon may be explained by the “original antigenic sin” effect, wherein vaccination with the ancestral strain preferentially recalls and expands memory B cells specific to the original virus [22]. Although the antibodies produced by these cells are high in titer, they exhibit reduced neutralizing efficiency against the highly mutated BF.7 and EG.5 variants. Furthermore, different routes of SARS-CoV-2 exposure differentially shape the B cell receptor (BCR) repertoire. Following natural infection, immunoglobulin (Ig) G1/3 and IgA1 BCRs increase, which activate a broad distribution of SARS-CoV-2-specific clones targeting the spike protein. In contrast, vaccination leads to an increased proportion of IgD/IgM BCRs and triggers a more focused response [23]. Thus, during natural infection, the antibody response is directly triggered by the infecting variant, leading to the production of specific antibodies with higher affinity for that particular strain. This may account for the comparable levels of effective NAbs against the homologous infecting variant observed between vaccinated and unvaccinated groups.

Notably, the recently circulating JN.1 variant poses a potential threat to existing hybrid immunity [24,25], underscoring the need for continuous monitoring of viral evolution and optimization of vaccine design. Recent studies have indicated that repeated antigen exposure is critical to attenuating immune imprinting induced by previous ancestral strain exposure and promoting robust specific antibody responses [26,27]. This study found that the hybrid immunity resulting from vaccination combined with natural infection, significantly enhances NAb levels and broadens antigen coverage, further validating the importance of multiple immune exposures in responding to viral evolution. However, vaccines currently designed based on the ancestral SARS-CoV-2 failed to induce detectable NAbs against the XBB sublineages (*p* < 0.05) (Figure 2), indicating that previous vaccines were unable to elicit a broad immune response that would have targeted the XBB variants. There is a need to develop new vaccines targeting the XBB serotype to achieve broad-spectrum protection [11].

As the core effector molecule of the humoral immune response, IgG typically emerges during the mid to late stages of infection. Characterized by a long half-life, it provides long-term protection and constitutes a critical component of immunological memory. Currently, the S protein of the ancestral strain serves as the basis for the majority of IgG detection reagents that are often employed in clinical and laboratory settings. Through the detection of antibody binding capabilities, these reagents provide an indirect indication of the host’s immunological state. However, determining the true level of immune protection requires figuring out the neutralizing potency of IgG antibodies, or their real capacity to prevent viral infection. This evaluation shows the antibodies’ ability to attach to antigens as well as their direct demonstration of functional neutralizing activities.

We systematically evaluated the relationship between IgG and NAb levels in the EG.5 infection cohort, revealing a significant positive correlation (Spearman r = 0.50–0.94, *p* < 0.01; Figure 4). However, this correlation strength varied substantially based on antigenic divergence. While pre-Omicron variants showed strong IgG-NAb concordance (r = 0.89–0.94), consistent with prior findings [28]. Omicron sublineages exhibited markedly weaker correlations (r = 0.50–0.55). This discordance likely reflects the cumulative impact of RBD mutations in Omicron subvariants, which alter critical epitopes while preserving sufficient structural similarity for antibody binding but not neutralization. Our data support the concept of ‘antigenic binding-neutralization dissociation’ [16,17], wherein immune imprinting from prior ancestral strain exposure generates cross-reactive antibodies with diminished neutralizing potency against divergent variants [29].

Consequently, conventional IgG detection assays, which are predominantly designed against the ancestral strain, exhibit considerable limitations in light of rapid viral evolution. Diminished epitope concordance can result in the overestimation of functional immunity (e.g., IgG-positive individuals with low neutralizing antibody levels) or the underestimation of protection (e.g., high neutralization titers in IgG-negative subjects) when testing against emerging variants. Our findings suggest that commercial IgG assays against the ancestral strain may overestimate the population’s actual immunity to currently circulating variants, indicating that serological surveillance data warrant a more cautious interpretation in public health decision-making. This dissociation highlights the urgent need to update antigen designs to match circulating variants in order to improve the reliability of clinical immunoassays, as well as the insufficiency of current serological instruments in accurately reflecting immune protection against divergent strains.

Even though previous research has shown a strong correlation between the severity of COVID-19 and the humoral immune response, the exact cause of this association has not yet been established. The overproduction of antibodies, which may be a sign of the severity of the disease, may be caused by severe illness, which is fueled by excessive inflammation and/or unchecked viral reproduction. This idea is supported by our findings, which reveal that elderly patients in critical condition have higher levels of NAbs and that there is a negative association between NAbs and LY and a positive correlation with inflammatory markers (WBC, NE, NLR, and CRP). This phenomenon can be explained from the perspective of virus-immune interaction mechanisms: on one hand, controlling viral replication requires strong immune activation, including pro-inflammatory cytokine release and immune cell proliferation. Increased inflammatory responses (seen by greater WBC, NE, NLR, and CRP) and lymphocyte depletion (lower LY) are frequently associated with high viral loads [30,31]. In contrast, extrapulmonary organ damage, including damage to the heart and kidneys, is often linked to systemic pro-inflammatory states, which are prevalent in severe COVID-19. The underlying mechanism might be a combination of two pathogenic processes: hyperactivated local inflammatory responses that cause oxidative stress cascades and mitochondrial malfunction, which in turn cause apoptosis, and direct viral invasion of cardiac and renal tissues via ACE2. It has been shown that some biomarkers of cardiac damage and dysfunction, like troponin and creatine kinase, can forecast clinical decline and worse outcomes in hospitalized patients [32].

The activity of LDH isoenzymes 1 and 2 (LDH-1 and LDH-2) is mostly reflected by HBDH, an isoenzyme of LDH. These isoenzymes are abundantly expressed in myocardial tissue, red blood cells, and kidneys, where they participate in the lactate–pyruvate conversion to maintain energy homeostasis. Elevated LDH levels have been frequently observed in many patients infected with SARS-CoV-2 and are utilized for predicting disease progression [33]. Changes in levels of HBDH can indicate cellular metabolic status and the extent of tissue damage, especially concerning cardiac and/or renal dysfunction. High HBDH concentrations are substantially linked to COVID-19 severity and mortality, according to meta-analyses, confirming its use as a crucial biomarker for risk classification [34]. The associations of neutralizing antibody levels with HBDH and LDH, considered alongside the comorbidity of coronary heart disease, collectively point to the necessity of closely monitoring renal health in the geriatric population after infection [35,36]. Taken together, the concurrent elevation of NLR and WBC in older patients points to a heightened systemic inflammatory response. Through the modification of inflammatory networks, the NAb response is thought to increase the risk of multi-organ damage [37], offering a novel perspective for risk assessment in elderly patients with COVID-19.

Clustering analysis of NAb responses in an elderly infected cohort further elucidates key features of viral evolution: SARS-CoV-2 variants can be divided into two major clades—pre-Omicron variants (including Gamma, D614G, Alpha, Beta, Kappa, and Delta) and Omicron variants (such as BA.1, BA.2, BA.2.75, BA.3, BA.4, BA.5, BF.7, BQ.1.1, XBB, and XBB.1.5)—with the Omicron lineage further differentiating into characteristic sublineages like XBB. This clustering pattern aligns with the natural trajectory of viral evolution and suggests a significant association between genetic features and pathogenicity [11,38]. More importantly, these results provide a rational basis for vaccine design: the antigenic convergence within the XBB lineage underscores its shared immune escape mechanisms. Therefore, next-generation vaccines should consider incorporating representative strains from the XBB lineage to improve cross-protective efficacy against emerging variants [39,40].

## 5. Conclusions

In summary, this study offers valuable insights for improving clinical management in elderly patients with COVID-19. Longitudinal monitoring of NAb levels—particularly cross-neutralizing activity against circulating variants—may facilitate early identification of high-risk individuals and help guide immunomodulatory treatment strategies. Concurrently, comprehensive interventions addressing risk factors such as obesity should be emphasized. However, several limitations remain: (1) the sample size was relatively small, particularly in the EG.5 severe group, necessitating larger multi-center studies to validate the generalizability of the findings; (2) the study did not incorporate cellular immune parameters such as T-cell responses, warranting integrated analyses with multi-dimensional immunological data to clarify underlying mechanisms; (3) the lack of longitudinal tracking of NAb dynamics (e.g., trends at 7, 14, and 28 days post-infection) highlights the need for future studies to establish their predictive utility. A comprehensive integration of virological, immunological, and clinical phenotypic data will be essential to underpin the development of precise prevention and clinical management strategies for elderly patients with COVID-19.

## Figures and Tables

**Table 1:** Demographic characteristics of the enrolled patients.

Characteristic		BF.7 Infection (*n* = 65)	EG.5 Infection (*n* = 57)
Sex			
	male	43	26
	female	22	31
Age [m (P25, P75)]		70.00 (65.00, 74.00)	72.00 (68.00, 80.00)
Vaccination			
	0	23	16
	1	0	0
	2	9	8
	3	15	11
	4	0	9
	unknown	18	13
Disease severity			
	mild	6	17
	moderate	28	29
	severe	25	11
	critical	6	0
Disease duration (Days) ^1^ [m (P25, P75)]		15.00 (13.00, 20.00)	13.00 (7.00, 18.00)

^1^ The period from the symptom onset to the sample collection.

## Data Availability

The original contributions presented in the study are included in the article/Supplementary Materials. Further inquiries can be directed to the corresponding author.

## Supplementary Materials

The following supporting information can be downloaded at: https://www.mdpi.com/article/10.3390/vaccines13111107/s1, Figure S1: Comparison of NAb levels between severe and non-severe EG.5-infected patients; Table S1: Comparison of characteristics between severe and non-severe groups.

## Abbreviations

The following abbreviations are used in this manuscript:

SARS-CoV-2	Severe Acute Respiratory Syndrome Coronavirus 2
RBD	Receptor-binding domain
S	Spike
ACE2	Angiotensin-converting enzyme 2
NAb	neutralizing antibody
bNAb	broad neutralizing-antibody
SA-PE	streptavidin–phycoerythrin
MFI	median fluorescence intensity
BMI	body mass index
WBC	white blood cell count
NE	neutrophil count
LY	lymphocyte count
NLR	neutrophil-to-lymphocyte ratio
CRP	C-reactive protein
LDH	lactate dehydrogenase
HBDH	hydroxybutyrate dehydrogenase
ICU	Intensive Care Unit

## References

[1] Wang Y., So H.C., Tsang N.N.Y., Kwok S.K., Cowling B.J., Leung G.M., Ip D.K.M. (2025). Clinical profile analysis of SARS-CoV-2 community infections during periods with omicron ba.2, ba.4/5, and XBB dominance in Hong Kong: A prospective cohort study. Lancet Infect. Dis..

[2] Markov P.V., Katzourakis A., Stilianakis N.I. (2022). Antigenic evolution will lead to new SARS-CoV-2 variants with unpredictable severity. Nat. Rev. Microbiol..

[3] Yang Y., Yang M., Peng Y., Liang Y., Wei J., Xing L., Guo L., Li X., Li J., Wang J. (2022). Longitudinal analysis of antibody dynamics in COVID-19 convalescents reveals neutralizing responses up to 16 months after infection. Nat. Microbiol..

[4] Zinatizadeh M.R., Zarandi P.K., Ghiasi M., Kooshki H., Mohammadi M., Amani J., Rezaei N. (2023). Immunosenescence and inflamm-ageing in COVID-19. Ageing Res. Rev..

[5] Gaspar Z., Szabo B.G., Andrikovics H., Cegledi A., Rajmon M., Abraham A., Varnai Z., Kiss-Dala N., Szlavik J., Sinko J. (2024). Secondary infections and long-term outcomes among hospitalized elderly and non-elderly patients with severe acute respiratory syndrome coronavirus 2 (SARS-CoV-2) and treated with baricitinib: A comparative study from the national centre of hungary. Geroscience.

[6] Collier D.A., Ferreira I.A.T.M., Kotagiri P., Datir R.P., Lim E.Y., Touizer E., Meng B., Abdullahi A., Elmer A., Kingston N. (2021). Age-related immune response heterogeneity to SARS-CoV-2 vaccine bnt162b2. Nature.

[7] Li J., Hui A., Zhang X., Yang Y., Tang R., Ye H., Ji R., Lin M., Zhu Z., Tureci O. (2021). Safety and immunogenicity of the SARS-CoV-2 bnt162b1 mRNA vaccine in younger and older Chinese adults: A randomized, placebo-controlled, double-blind phase 1 study. Nat. Med..

[8] Levin E.G., Lustig Y., Cohen C., Fluss R., Indenbaum V., Amit S., Doolman R., Asraf K., Mendelson E., Ziv A. (2021). Waning immune humoral response to bnt162b2 COVID-19 vaccine over 6 months. N. Engl. J. Med..

[9] Tartof S.Y., Slezak J.M., Fischer H., Hong V., Ackerson B.K., Ranasinghe O.N., Frankland T.B., Ogun O.A., Zamparo J.M., Gray S. (2021). Effectiveness of mRNA bnt162b2 COVID-19 vaccine up to 6 months in a large integrated health system in the USA: A retrospective cohort study. Lancet.

[10] Zhang X., Wang Y., Li M., Li H., Zhang X., Xu X., Ma Q., Hu D., Jia Y., Liang T. (2025). A High-Throughput Broad Neutralizing Antibody Assay for Detecting SARS-CoV-2 Variant Immunity in Population. ACS Infect. Dis..

[11] Li Y., Zhang X., Yi J., Chen Y., Liang J., Wang L., Ma J., Zhu R., Zhang X., Hu D. (2024). Synergistic evolution: The dynamic adaptation of SARS-CoV-2 and human protective immunity in the real world. J. Infect..

[12] Pan Y., Wang L., Feng Z., Xu H., Li F., Shen Y., Zhang D., Liu W.J., Gao G.F., Wang Q. (2023). Characterisation of SARS-CoV-2 variants in Beijing during 2022: An epidemiological and phylogenetic analysis. Lancet.

[13] Dyer O. (2023). COVID-19: Infections climb globally as eg.5 variant gains ground. BMJ.

[14] Wang Q., Iketani S., Li Z., Liu L., Guo Y., Huang Y., Bowen A.D., Liu M., Wang M., Yu J. (2023). Alarming antibody evasion properties of rising SARS-CoV-2 BQ and XBB subvariants. Cell.

[15] Li Y., Chen Y., Liang J., Wang Y. (2024). Immunological characteristics in elderly COVID-19 patients: A post-COVID era analysis. Front. Cell. Infect. Microbiol..

[16] Cao Y., Wang J., Jian F., Xiao T., Song W., Yisimayi A., Huang W., Li Q., Wang P., An R. (2022). Omicron escapes the majority of existing SARS-CoV-2 neutralizing antibodies. Nature.

[17] Liu L., Iketani S., Guo Y., Chan J.F., Wang M., Liu L., Huang W., Li Q., Wang P., An R. (2022). Striking antibody evasion manifested by the omicron variant of SARS-CoV-2. Nature.

[18] Legros V., Denolly S., Vogrig M., Boson B., Siret E., Rigaill J., Pillet S., Grattard F., Gonzalo S., Verhoeven P. (2021). A longitudinal study of SARS-CoV-2-infected patients reveals a high correlation between neutralizing antibodies and COVID-19 severity. Cell Mol. Immunol..

[19] Jager M., Sonnleitner S.T., Dichtl S., Lafon E., Diem G., Walder G., Lass-Florl C., Wilflingseder D., Posch W. (2022). Immune responses against SARS-CoV-2 WT and delta variant in elderly bnt162b2 vaccinees. Front. Immunol..

[20] Lopez Bernal J., Andrews N., Gower C., Robertson C., Stowe J., Tessier E., Simmons R., Cottrell S., Roberts R., O’Doherty M. (2021). Effectiveness of the Pfizer-BioNTech and Oxford-AstraZeneca vaccines on COVID-19 related symptoms, hospital admissions, and mortality in older adults in England: Test negative case-control study. BMJ.

[21] Shrotri M., Krutikov M., Palmer T., Giddings R., Azmi B., Subbarao S., Fuller C., Irwin-Singer A., Davies D., Tut G. (2021). Vaccine effectiveness of the first dose of chadox1 ncov-19 and bnt162b2 against SARS-CoV-2 infection in residents of long-term care facilities in England (VIVALDI): A prospective cohort study. Lancet Infect. Dis..

[22] Gao B., He L., Bao Y., Chen Y., Lu G., Zhang Y., Xu Y., Su B., Xu J., Wang Y. (2023). Repeated vaccination of inactivated SARS-CoV-2 vaccine dampens neutralizing antibodies against omicron variants in breakthrough infection. Cell Res..

[23] Kotagiri P., Mescia F., Rae W.M., Bergamaschi L., Tuong Z.K., Turner L., Hunter K., Gerber P.P., Hosmillo M., Hess C. (2022). B cell receptor repertoire kinetics after SARS-CoV-2 infection and vaccination. Cell Rep..

[24] Jian F., Wang J., Yisimayi A., Song W., Xu Y., Chen X., Niu X., Yang S., Yu Y., Wang P. (2025). Evolving antibody response to SARS-CoV-2 antigenic shift from XBB to jn.1. Nature.

[25] Gulova S.M., Veselkina U.S., Astrakhantseva I.V. (2025). Adaptation of the vaccine prophylaxis strategy to variants of the SARS-CoV-2 virus. Vaccines.

[26] Yisimayi A., Song W., Wang J., Jian F., Yu Y., Chen X., Xu Y., Yang S., Niu X., Xiao T. (2024). Repeated omicron exposures override ancestral SARS-CoV-2 immune imprinting. Nature.

[27] Gong X., Peng L., Wang F., Liu J., Tang Y., Peng Y., Niu S., Yin J., Guo L., Lu H. (2024). Repeated omicron infection dampens immune imprinting from previous vaccination and induces broad neutralizing antibodies against omicron sub-variants. J. Infect..

[28] Wajnberg A., Amanat F., Firpo A., Altman D.R., Bailey M.J., Mansour M., McMahon M., Meade P., Mendu D.R., Muellers K. (2020). Robust neutralizing antibodies to SARS-CoV-2 infection persist for months. Science.

[29] Roltgen K., Nielsen S.C.A., Silva O., Younes S.F., Zaslavsky M., Costales C., Yang F., Wirz O.F., Solis D., Hoh R.A. (2022). Immune imprinting, breadth of variant recognition, and germinal center response in human SARS-CoV-2 infection and vaccination. Cell.

[30] Rao S.N., Manissero D., Steele V.R., Pareja J. (2020). A systematic review of the clinical utility of cycle threshold values in the context of COVID-19. Infect Dis Ther..

[31] Fajnzylber J., Regan J., Coxen K., Corry H., Wong C., Rosenthal A., Worrall D., Giguel F., Piechocka-Trocha A., Atyeo C. (2020). SARS-CoV-2 viral load is associated with increased disease severity and mortality. Nat. Commun..

[32] Shi S., Qin M., Cai Y., Liu T., Shen B., Yang F., Cao S., Liu X., Xiang Y., Zhao Q. (2020). Characteristics and clinical significance of myocardial injury in patients with severe coronavirus disease 2019. Eur. Heart J..

[33] Shcherbak S.G., Anisenkova A.Y., Mosenko S.V., Glotov O.S., Chernov A.N., Apalko S.V., Urazov S.P., Garbuzov E.Y., Khobotnikov D.N., Klitsenko O.A. (2021). Basic predictive risk factors for cytokine storms in COVID-19 patients. Front. Immunol..

[34] Zinellu A., Paliogiannis P., Carru C., Mangoni A.A. (2022). Serum hydroxybutyrate dehydrogenase and COVID-19 severity and mortality: A systematic review and meta-analysis with meta-regression. Clin. Exp. Med..

[35] Boulos P.K., Freeman S.V., Henry T.D., Mahmud E., Messenger J.C. (2023). Interaction of COVID-19 with common cardiovascular disorders. Circ. Res..

[36] Zhang J., Pang Q., Zhou T., Meng J., Dong X., Wang Z., Zhang A. (2023). Risk factors for acute kidney injury in COVID-19 patients: An updated systematic review and meta-analysis. Ren. Fail..

[37] Garcia-Beltran W.F., Lam E.C., Astudillo M.G., Yang D., Miller T.E., Feldman J., Hauser B.M., Caradonna T.M., Clayton K.L., Nitido A.D. (2021). COVID-19-neutralizing antibodies predict disease severity and survival. Cell.

[38] Zhang X., Li M., Zhang N., Li Y., Teng F., Li Y., Zhang X., Xu X., Li H., Zhu Y. (2024). SARS-CoV-2 evolution: Immune dynamics, omicron specificity, and predictive modeling in vaccinated populations. Adv. Sci..

[39] Yang J., He X., Shi H., He C., Lei H., He H., Yang L., Wang W., Shen G., Yang J. (2025). Recombinant xbb.1.5 boosters induce robust neutralization against kp.2- and kp.3-included jn.1 sublineages. Signal Transduct. Target. Ther..

[40] Yang G., Lu M., Chen R., Wang S., Wan S., Song X., Cao G., Lv L., He X., Zhan B. (2025). Neutralizing antibody responses to three XBB protein vaccines in older adults. Signal Transduct. Target. Ther..

