# Supplementary material for: Neutralizing Antibody Response Characteristics in Elderly Patients with SARS-CoV-2 Infection and Their Association with Clinical Phenotypes
Source: Vaccines (Basel). 2025 Oct 29;13(11):1107. doi: 10.3390/vaccines13111107 (PMC12656855; doi:10.3390/vaccines13111107)
Supplement: Supplementary file 1 [file vaccines-13-01107-s001.zip › vaccines-3906178-supplementary.pdf]

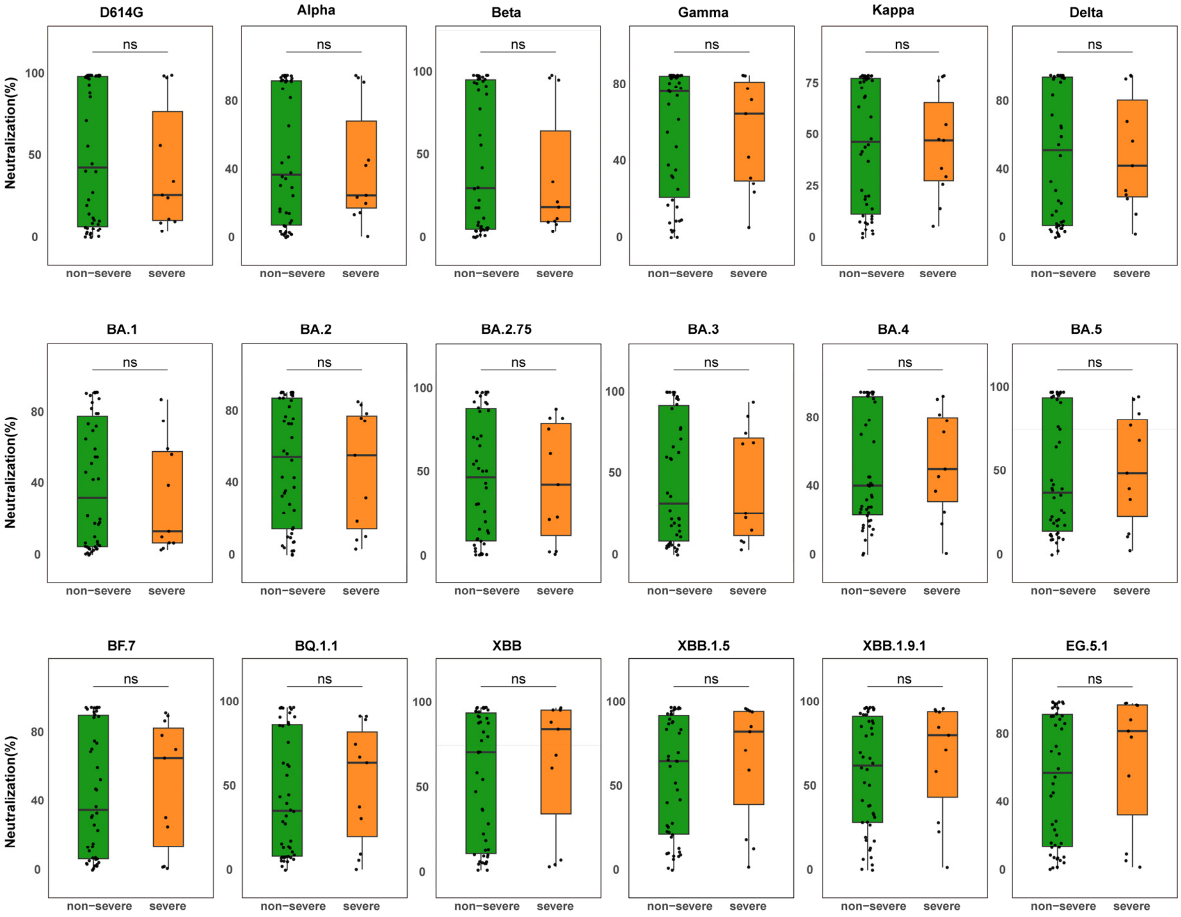

Supplementary Figure S1. Comparison of NAb levels between severe and non-severe EG.5-infected patients. Box plots show NAb levels against the SARS-CoV-2 variant S protein in non-severe patients (green, *n* = 46) and severe patients (orange, *n* = 11). The boxes represent the median (center line) and interquartile range (25-75%). * *p* < 0.05, ** *p* < 0.01, ns: not significant.

Supplementary Table S1. Comparison of characteristics between severe and non-severe groups.

**Characteristic**		**Non-severe (*n* = 60)**	**Severe (*n* = 31)**	***P***
Age [m(P25, P75)]		69[66,75.2]	74[69.5,80.5]	0.033*
Sex				0.001**
	male	25(42%)	24(77%)	
	female	35(58%)	7(23%)	
Vaccination	0	22(37%)	15(48%)	0.3
	≥1	38(63%)	16(52%)	
Comorbidity				0.047*
	yes	24(40%)	6(19%)	
	no	36(60%)	25(81%)	

**P* < 0.05, ***P* < 0.01
